# Radiological long-term follow-up up to 12 years of initially ultrasound unstable hip types D, III and IV after treatment with the Tübingen splint worn as a plaster

**DOI:** 10.1007/s00402-023-04807-z

**Published:** 2023-02-21

**Authors:** Hannes Kubo, Ruediger Krauspe, Hakan Pilge, Bettina Westhoff

**Affiliations:** 1 Medical Faculty, Department of Orthopaedics and Trauma Surgery, University of Duesseldorf, Moorenstr. 5, 40225 Duesseldorf, Germany; 2 Orthopaedicum Munich, Charles-de-Gaulle-Str. 4, 81737 Munich, Germany

**Keywords:** Developmental dysplasia of the hip (DDH), Tübingen splint, Unstable hips, Radiological follow-up

## Abstract

**Introduction:**

The treatment of ultrasound unstable hips with the Tübingen splint is currently under discussion. However, there is a lack of long-term follow-up data. This study presents to the best of our knowledge first radiological mid-term to long-term data of the successful initial treatment with the Tübingen splint of ultrasound unstable hips.

**Materials and methods:**

From 2002 to 2022 the treatment of ultrasound unstable hips type D, III and IV (age ≤ 6 weeks, no severe limitation of abduction) with the Tübingen splint worn as a plaster is evaluated. Based on data derived from routine X-rays during the follow-up period, a radiological follow-up (FU) analysis until the age of 12 years was performed. The acetabular index (ACI) and center–edge angle (CEA) were measured and classified according to Tönnis as normal findings (NF), slightly (sliD) or severely dysplastic (sevD).

**Results:**

193 of 201 (95.5%) unstable hips could be successfully treated showing normal findings with an alpha angle > 65°. The few patients showing treatment failures were successfully treated applying a Fettweis plaster (human position) under anesthesia. The radiological FU of 38 hips showed a favorable trend with increase of normal findings from 52.8% to 81.1% and decrease of 38.9% to 19.9% of sliD respectively 8.3% to 0% of sevD hips. The analysis of avascular necrosis of the femoral head showed 2 cases (5.3%) of grade 1 according to Kalamchi and McEwen, which were improving over time in the further course.

**Conclusions:**

The Tübingen splint as alternative to replace a plaster has proven a successful therapeutic option for ultrasound unstable hips type D, III and IV with favorable and over time improving radiological parameter up to the age of 12 years.

## Introduction

Developmental dysplasia of the hip (DDH) is the most common disease of hip joints in childhood and therefore a major clinical and scientific focus for pediatric orthopedists. DDH is characterized by a dysplastic maturation disorder of the lateral edge of the acetabulum and, associated with this, a lack of coverage of the acetabular roof over the femoral head [1]. During further development, the femoral head may dislocate resulting in an increase of pressure on the already dysplastic lateral edge and its growth area of the acetabulum leading to a further impairment of the acetabular development. Symptoms of DDH like pain and/ or limitation of the range of motion most often only occur with structural damage at a later time. Clinical signs and tests such as the Ortolani and Barlow tests are often unspecific (especially in borderline cases) or can only be properly performed by experienced examiners [2]. The implementation of the hip ultrasound examination in the 1980s by Graf and the introduction of a screening program in several countries led to a fundamental improvement for the children with a significant earlier diagnosis and less invasive as well as shorted treatment [3–7].

Based on the ultrasound characteristics dysplastic hips can be classified into ultrasound stable and unstable hips. Ultrasound stable hips (hip type IIa, IIb and IIc according to Graf) are treated with so-called maturation orthoses such as the Tübingen splint or others; ultrasound unstable hips (hip type IID, III and IV) have to be securely reduced and after stabilization to be treated with retention devices such as the Fettweis plaster or Pavlik harness [8, 9]. In recent studies, the Tübingen splint is currently applied for the treatment of ultrasound unstable hips—especially in newborns up to the age of 6 to 8 weeks: it was shown that 92.3% to 98.0% of the unstable hips could be transferred into an ultrasound normal hip with the Tübingen splint [10–12]. Advantages of the Tübingen splint are easy handling, easy adjustment in size and easy cleaning.

After successful therapy, the hips need radiological controls until cessation of growth in order to detect residual or recurrent dysplastic changes at an early stage and to reveal complications such as avascular necrosis of the femoral head (AVN). Therefore, an X-ray of the pelvis is an established method for follow-up examinations. By analyzing these radiographs, the bony development can be observed using several lines and angles, such as the acetabular index (ACI) and the center edge angle (CEA). In adolescents the ACI is no longer valid due to the closing of the triradiate epiphyseal plates. Here, the CEA is an established method to assess residual dysplasia.

Tönnis et al. analyzed and classified the development of the normal and abnormal hips. Based on these results, hips can be classified into normal findings, slightly or severely dysplastic pathologic findings [13]. The classification depends on the ACI, gender, affected side and age of the individual. Based on the classification therapy, recommendations can be made.

Until now, there are only limited data on the short- and mid-term radiological development of ultrasound unstable hips after treatment with the Tübingen splint [14]. Data on long-term outcome are not available yet.

Therefore, the aim of the present study is to evaluate the success of the treatment of ultrasound unstable hips with the Tübingen splint. Furthermore, the radiographic long-term development up to the age of 12 years will be analyzed.

## Material and methods

### Patients and ultrasound examinations

We examined patients with ultrasound unstable hips type D, III and IV according to Graf, which were treated with the Tübingen splint in the period from 2002 to 2022. The parents were advised to wear the splint like a plaster (strictly no discontinuation for daily care) until the hip is transferred in an ultrasound stable hip (type IIa, IIb, IIc-stable). Inclusion criteria for treatment were (1) initiation of treatment ≤ 6 weeks of life and (2) no severe limitation of range of motion especially for abduction. Patients with secondary dysplasia—for example due to neurological impairment—were excluded.

**Fig. 1:**
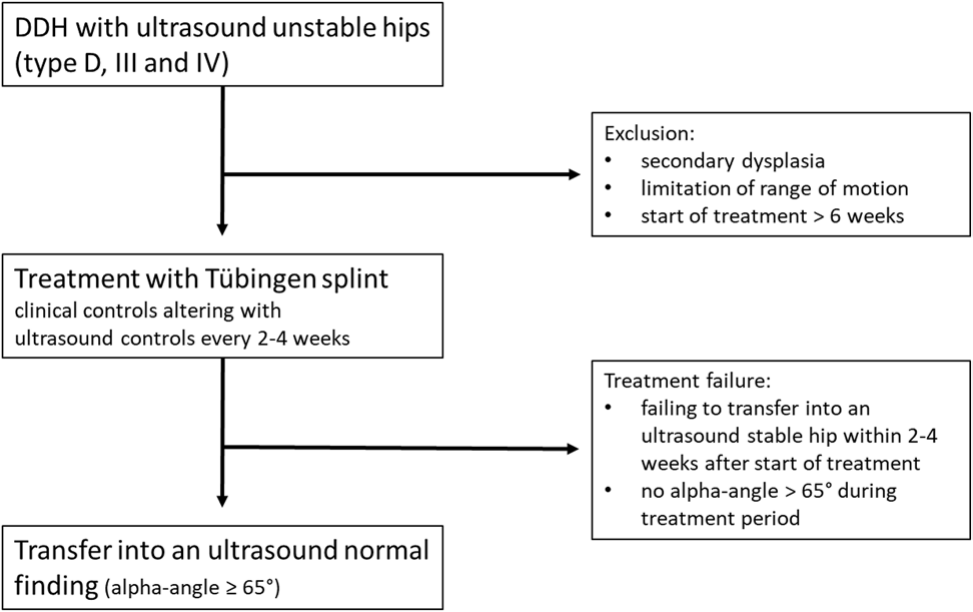
Treatment protocol of ultrasound unstable hips type D, III and IV with Tübingen splint

The evaluated parameters were (1) average age at beginning of the treatment, (2) average duration of treatment (3) initial ultrasound hip type according to Graf, (4) affected side and (5) gender. Endpoint of this part of the study was defined as normalization of the hip showing an alpha-angle ≥ 65° (Fig. 1).

Part of the data of this study group were already analyzed and published previously [10].

### Radiological examinations

From the above-mentioned group, a radiological follow-up (FU) until the age of 12 years was performed. Inclusion criteria for this second part of the study were (1) successful treatment of ultrasound unstable hips type D, III and IV with the Tübingen splint with transfer into ultrasound normal findings and (2) the presence of radiographs at the age of 8–12 years with at least 3 earlier radiographs at the age of 1–2, 2–4 years and/or 4–8 years.

Based on these radiographs, the acetabular index (ACI, in the first three radiographs) and the center edge angle (CEA, in the last two radiographs) were determined. According to the Tönnis classification, the measured radiological parameters were classified into (1) normal findings, (2) slightly dysplastic hips and (3) severely dysplastic hips [13]. Additionally, the hips were analyzed for the development of an avascular necrosis of the femoral head (AVN) according to Kalamchi and McEwen [15]. Likewise, we investigated whether a surgical treatment was recommended or performed.

### Statistical analysis

The data were analyzed using the statistic program SPSS 25 (IBM Corp., Armok, New York). The statistical analysis was done using Student’s t test. A *p*-value ≤ 0.05 was considered as statistically significant.

## Results

### Patients

We could include 147 patients with 201 (54 bilateral) ultrasound unstable hips type D, III and IV. The initial ultrasound hip type was in 93 cases type D (46.3%), in 86 cases type III (42.8%) and in 22 cases type IV (10.9%). One-hundred twenty-nine patients (87.8%) were female, and the left side was affected more frequently (117/201) (58.2%). Out of this group, 25 patients with 38 (13 bilateral) initially ultrasound unstable hips could be followed radiologically up to 12 years with a minimum FU of 8 years.

### Ultrasound examinations

**Fig. 2:**
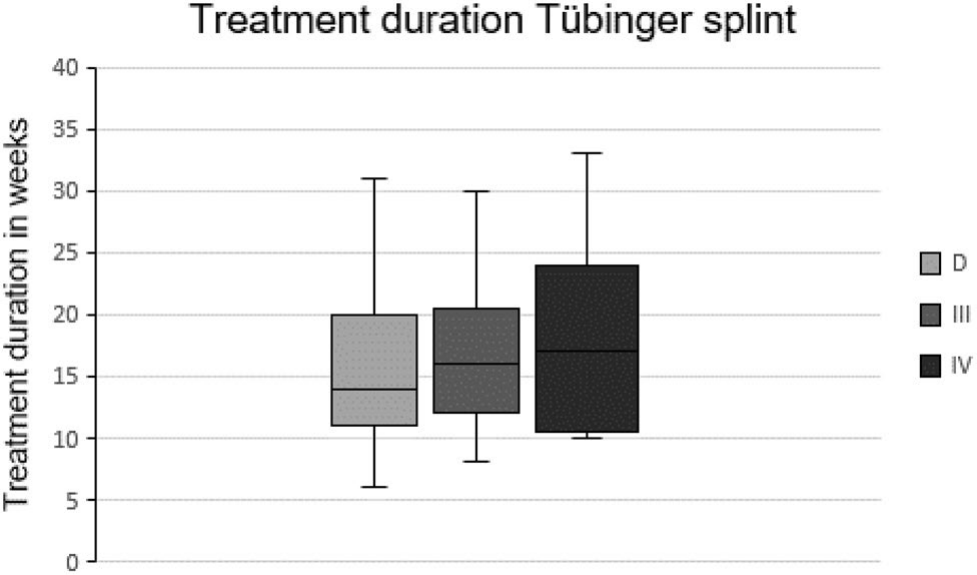
Treatment duration of ultrasound unstable hips with the Tübingen splint in weeks, *n* = 192

**Fig. 3:**
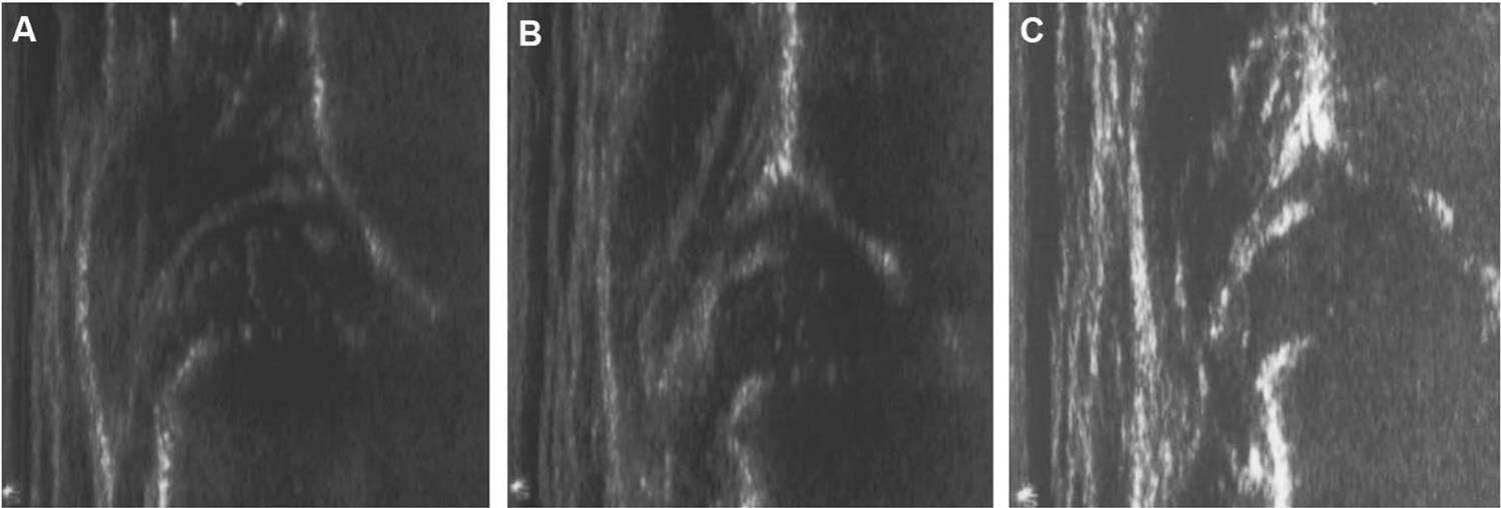
Ultrasound examination of female, right side: **A** initial ultrasound examination at the age of 41 days with an ultrasound hip type IV and start of treatment with the Tübingen splint, **B** ultrasound control after 3 weeks showing a centered hip with alpha angle of 46°, **C** successful treatment after 15 weeks with normal ultrasound characteristics and an alpha angle of 67°

A total of 192 of 201 ultrasound unstable hips (95.5%) were successfully treated with the Tübingen splint and could be transferred into an ultrasound normal finding with an average alpha-angle of 69.3° at latest ultrasound follow-up (SD ± 2.5, range 65–77°). The therapy failed in 9 hips (see treatment failures). The average start of treatment was at the age of 22.6 days (SD ± 14.2, range 0–42), and the average duration of treatment was 16.1 weeks (SD ± 5.8, range 6–33). There were slight differences when comparing the different ultrasound hip types but without statistical significance. The average duration of treatment of hip type D was 15.6 weeks (SD ± 5.6, range 6–31), of hip type III 16.4 weeks (SD ± 5.7, range 8–30) and of hip type IV 18.1 weeks (SD ± 7.2, range 10–33) (Figs. 2, 3).

### Treatment failures

Nine of 201 ultrasound unstable hips (4.5%) could not be transferred into an ultrasound normal finding: 4 out of 22 initially ultrasound hip type IV (18.8%), 4 of 86 hip type III (4.7%) and 1 of 93 hip type D (1.1%). Hip type III and IV showed also signs of instability on clinical examination. In all cases a closed reduction—verified by arthrography—and application of a Fettweis plaster in human position were performed which finally led to a successful treatment outcome resulting in an ultrasound type I hip during follow-up. Before discharge of the patient at this time of the course of therapy, the reduction of the femoral head was controlled by MRI in all cases. There was no need for an open reduction in any case. The one affected hip type D is one of bilateral DDH in which the other hip was a hip type IV, which needed a treatment conversion to closed reduction and retention in a Fettweis plaster. Thus, an exact statement about a potential therapy failure of this type D hip is not possible.

### Radiological examinations

Thirty-eight initially ultrasound unstable hips type D, III and IV could be included in the radiological long-term FU (type D: 13 (34.2%), type III 20 (52.6%), type IV 5 (13.2%). The average age at the first radiograph was 13.7 months (1.1 years, SD ± 1.4, range 11–17 months), for the second radiograph 32.3 months (2.7 years, SD ± 6.9, range 23–48 months), for the third 69.2 months (5.8 years, SD ± 13.0, range 50–91 months) and at the fourth radiograph 118.7 months (9.9 years, SD ± 10.4, range 99–152 months).

**Fig. 4:**
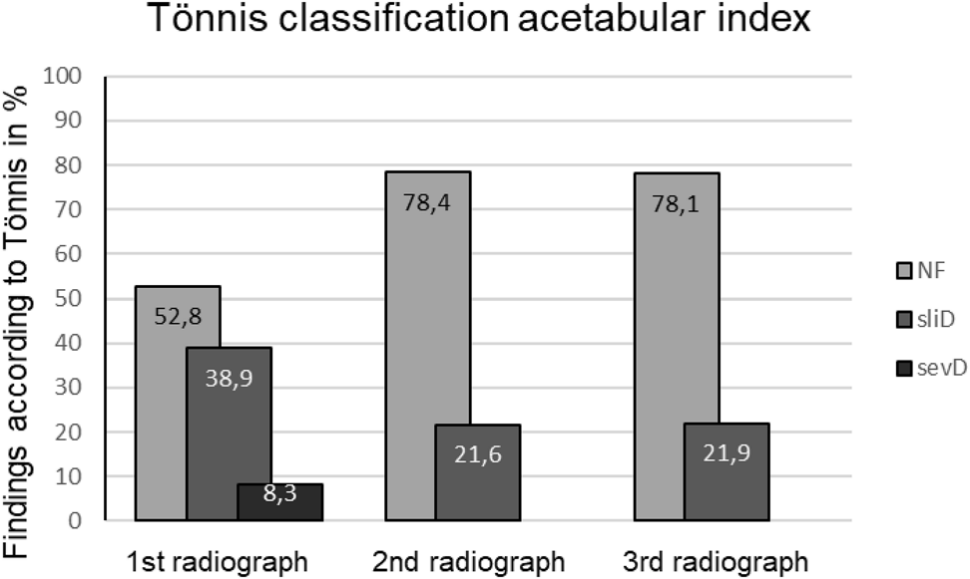
Classification according to Tönnis of the ACI of the first, second and third radiograph, *NF* normal finding, *sliD* slightly dysplastic findings, *sevD* severely dysplastic findings in percent

**Fig. 5:**
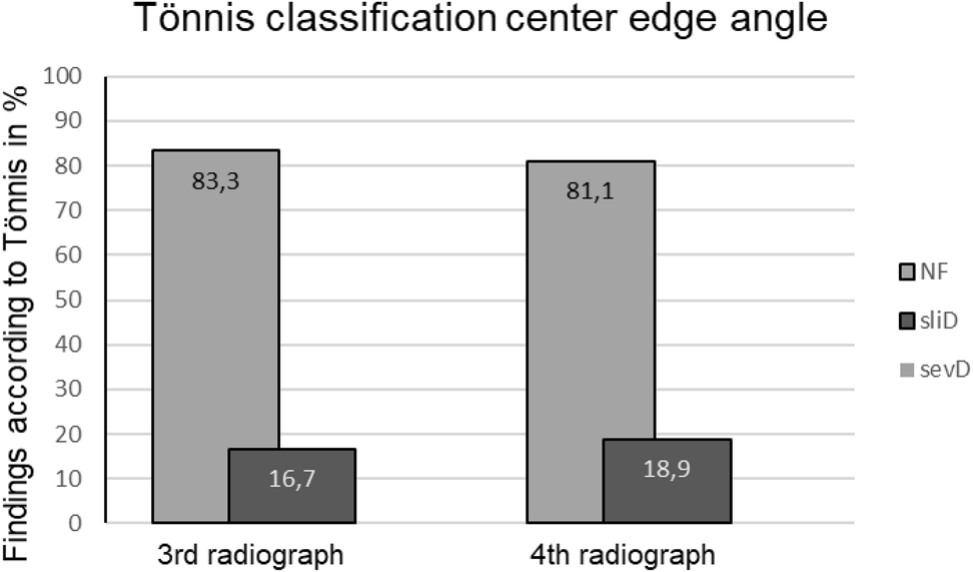
Classification according to Tönnis of the CEA of the third and fourth radiograph, *NF* normal finding, *sliD* slightly dysplastic findings, *sevD* severely dysplastic findings in percent

**Fig. 6:**
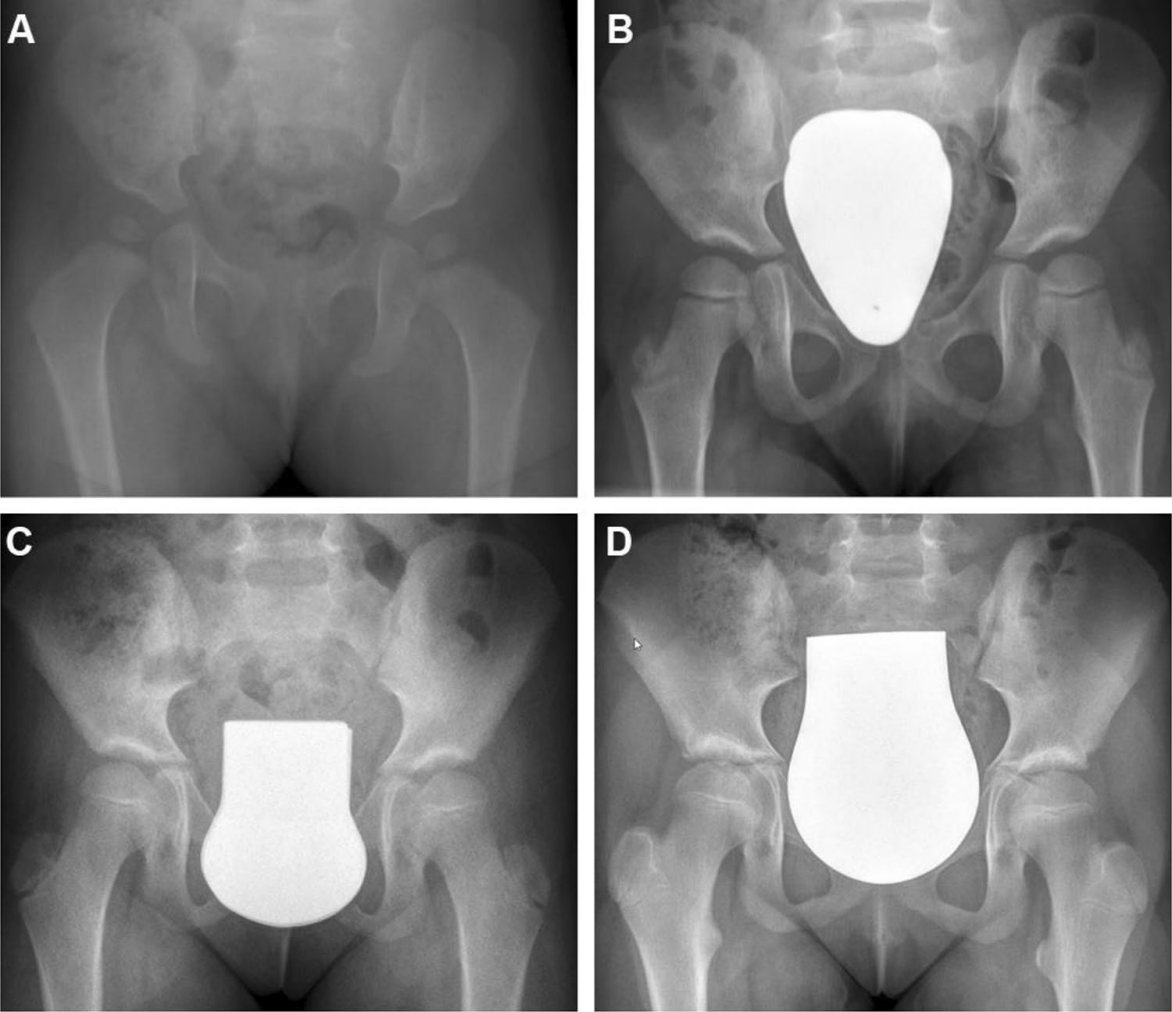
Radiological development of the patient of Fig. 3 with an initial hip type IV on the right side, **A** X-ray control at the age of 15 months: ACI: 26° (Tönnis classification: slightly dysplastic), **B** at the age of 48 months: ACI: 21° (Tönnis classification: normal finding), **C** at the age of 80 months: ACI: 18°, CEA 28° (Tönnis classification: normal finding), **D** at the age of 124 months: ACI: 11°, CEA: 30° (Tönnis classification: normal finding)

The analysis of the ACI showed a favorable evolution of the hips with increase of normal findings (52.8–78.1% from the first to fourth radiograph) and a decrease of slightly and severely dysplastic hips (38.9–21.9% and 8.3–0%, respectively, from the first to the fourth radiograph) (Fig. 4). This positive development occurred especially between the first and second radiograph. In the further course the level remained consistently good up to the fourth radiograph. Here the examination of the CEA showed constant high levels of normal findings (> 80%) and low levels of slightly pathologic hips (< 20%) (Figs. 5, 6). Severely dysplastic hips were not detected at latest FU (Table 1).

**Table 1:** ACI measurements at the first, second and third radiograph and CEA measurements at the third and fourth radiograph

	ACI 1	ACI 2	ACI 3	CEA 3	CEA 4
Mean value	25.2°	21.2°	16.5°	26.3°	29.7°
Median	25.0°	21.0°	16.0°	25.5°	29.8°
Average age at radiograph	1.1 ys	2.7 ys	5.8 ys	5.8 ys	9.9 ys
SD	3.2	3.2	4.1	6.3	5.2
Minimum	18°	13°	8°	18°	20°
Maximum	32°	28°	23°	39°	42°

When analyzing the acetabular development in dependence of the initial ultrasound hip type, the positive tendency up to the fourth radiograph could be found especially for the initially hip types D (75.0% normal findings) and type III (95.0% normal findings); thus, 25.0% (D) and 5.0% (III) slightly dysplastic hips were found. Initially, type IV hips showed a poorer development with only 40.0% normal findings and 60.0% slightly dysplastic hips.

### Improvements and deteriorations

During the follow-up period, an improvement of the hip according to the Tönnis classification could be observed in 18 hips, in 4 hips deterioration occurred (Table 2). Most of the improvements (72.2%) occurred in the period between the first and second radiograph—11.1% after the second and 16.6% after the third radiograph. Deteriorations also occurred predominantly between the first and second radiograph (75.0%), 25.0% after the second radiograph. In two cases (both initially type D), there were improvements and deteriorations within the follow-up period. Here, the fourth radiograph showed improvement in one case and deterioration in the other.

**Table 2:** Improvements and deteriorations by initial ultrasound hip type and time point of the radiograph

	Hip type D	Hip type III	Hip type IV
*Improvements*	7 (of 13,53.8%)	9 (of 20,45.0%)	2 (of 5,40%)
From radiograph 1 to 2	4	7	2
From radiograph 2 to 3	–	2	–
From radiograph 3 to 4	3	–	–
*Deteriorations*	3 (of 13,23.1%)	0	1 (of 5, 20%)
From radiograph 1 to 2	2	–	1
From radiograph 2 to 3	1	–	–
From radiograph 3 to 4	–	–	–

### Avascular necrosis of the femoral head

Two cases (5.3%) of avascular necrosis of the femoral head (AVN) were detected. Both cases were low-degree AVN type I according to Kalamchi and McEwen and showed a restitutio ad integrum during follow-up.

## Discussion

The analysis after treatment of ultrasound unstable hips type D, III and IV with the Tübingen splint and start of treatment below the age of 6 weeks shows a good success rate of 95.5% with transfer into an ultrasound normal finding with an alpha-angle above 65°. The value 65° is used with respect to the maturation curve of Tschauner et al. that postulated a higher alpha-angle in the further development of the hip joints in the first few months [16].

The above-mentioned success rate of 95.5% is comparable with previous studies, which examined the therapy of ultrasound unstable hips with the Tübingen splint. Here, success rates of 92.3–98.0% were observed [10–12, 17]. Seidl et al. found in their study with 52 ultrasound unstable hips a success rate of 98.0% [11]. Compared to our study they started treatment earlier—during the first week of life—and only one hip type IV was included. Pavone et al. were able to successfully treat 92.3% of ultrasound unstable hips with the Tübingen splint (*n* = 251) [12]. However, no specific information is provided about initial ultrasound hip type, average age at initiation of treatment and duration of treatment. Zhi et al. found in a meta-analysis a success rate for treatment with the Tübingen splint in hip type II (without precise differentiation of subtypes) of 98.0% and in type III hips of 96.0%. This is in accordance to our data with 98.9% success rate of type D and 95.3% success rate of type III hips. On the other hand, the success rate of 32.0% for type IV hips was very low (with statistically low heterogeneity in the study group) [17]. These data were significantly inferior to the results of the present study showing a success rate of 81.2% for type IV hips. This could be explained by the fact of inclusion of children younger than 6 weeks and without severe limitation of hip abduction in our study; the parents were also advised to wear the splint like a cast—meaning not to take off the splint at any time (24 h/d and 7d/w). A detailed comparison with the other studies is difficult because of the heterogenic of the different study population.

Compared to other treatment options for ultrasound unstable hips—such as the frequently used Fettweis plaster or the Pavlik harness—, the stated success rate is comparably good. The Fettweis plaster shows a success rate of 80 to 93% with a low AVN rate < 7.4% over time [18–21]. However, the Fettweis plaster is applied under anesthesia associated with a higher burden for the patient and it is less comfortable for the parents in taking care for hygiene, etc. Another alternative is the widely used Pavlik harness with a success rate of 73 to 92% [22, 23]. Disadvantages compared to the Tübingen splint are the higher AVN rate of up to 16.1% and the more challenging application, handling and cleaning of the baby for the parents [23–27]. Furthermore, the therapy duration up to six months is longer than with the Tübingen splint [28].

In the present study, the treatment failures with the Tübingen splint affected mainly hip types IV with 18.8% (4 of 22) compared to 1.1% (1 of 93) for hip type D and 4.7% (4 of 86) for hip type III. Therefore, treatment with the Tübingen splint should be carefully considered for the most severe type of hip dislocation. In favor for a therapeutic approach of hips, type IV with the Tübingen splint is the success rate of over 80%; in case of a failure to achieve a centered hip joint within the first 2 – 4 weeks after start of treatment, the modality will be converted to closed reduction and retention in a Fettweis plaster which then would be applied still timely within the first three months of life. The results show that finally all these cases could be successfully treated and transferred to an ultrasound hip type I. An open reduction was not required in any case. On the other hand, one might argue that the failed initial treatment would prolong the overall-treatment time, but in summary the over-all success-rate of 95.5% is promising and supports our initial approach.

The radiological follow-up until the age of 12 years showed a positive development of the hips with a rate of more than 80% normal findings and less than 20% slightly dysplastic hips at the latest follow-up X-ray (fourth radiograph). Most changes—predominantly improvements—were seen between the first and second radiographs taken at the time of 1–2 years and 2–4 years. Therefore, the question arises whether all hips require radiological controls at all time-points applied so far. However, the indication of radiological controls is supported by the fact that changes with improvements and deteriorations could be detected in all hip types at all time points. Especially type IV hips—due to the rather worse tendency—need regular controls which allow for less invasive surgical treatment during childhood in case of persistent or recurrent dysplasia. This can usually be performed by a single pelvic osteotomy while in adolescents or adults more complex types of surgery become necessary (triple pelvic osteotomy or periacetabular osteotomy). The concern of radiation exposure is diminished by the fact that modern X-ray technology with significant lower radiation exposure is used today after the introduction of digital radiography [29]. Here Vogel et al. showed a mean cumulative effective dose of 0.25 mSv of anterior–posterior pelvic radiographs. In comparison to that, the annual limit for healthcare workers is 20 mSv. The effective dose is classified as at very low risk [29]. In summary the current diagnostic and therapeutic approach shows a significant improvement in the detection and treatment of DDH: earlier and shorter treatment, less surgical interventions and less impairment of the physiological development of the affected child. The time points for X-ray examinations may better defined with more data of further studies with higher case numbers.

The rate of avascular necrosis of the femoral head (AVN) of 5.3% is similar to other treatment modalities such as Fettweis plaster with 2.1–7.4% and significantly lower compared to Pavlik harness with 2.4–16.1% [23–27]. In addition, the AVN cases in our study only showed the lowest grade 1 according to Kalamchi and McEwen, which resolved in the further course. Other studies showed a more heterogeneous outcome with AVN levels of 1 to 3 [24, 25]. The comparisons with other outcome studies are problematic due to different study designs, different study populations and different study protocols.

Limitations of this study are the retrospective study design and the relative low number of patients in the long-term FU. But these are the data available at this time, showing the longest follow-up in this study population. We conclude that the modalities are supported by the results.

## Conclusions

Treatment of ultrasound unstable hips type D, type III and type IV with the Tübingen splint which is worn like a plaster (no discontinuation at all) is a successful option especially for type D and III hips and—with a lower success rate—also for type IV hips. The treatment should start as early as possible, ideally before the age of 6 weeks and patients should not have any restrictions of range of motion specially of abduction. A favorable evolution could also be observed radiographically in the long-term follow-up to the age of 12 years. Nevertheless, regular radiological controls are recommended, since changes may occur and can be detected at the selected time point that then allows to indicate further treatment like corrective osteotomies in time.

## Data Availability

The datasets used and/or analyzed during the current study are available from the corresponding author on reasonable request.
